# The Impact of Non-attempted and Dually-Attempted Items on Person Abilities Using Item Response Theory

**DOI:** 10.3389/fpsyg.2016.01572

**Published:** 2016-10-14

**Authors:** Georgios D. Sideridis, Ioannis Tsaousis, Khaleel Al Harbi

**Affiliations:** ^1^Clinical Research Center, Boston Children’s Hospital, Harvard Medical School, BostonMA, USA; ^2^Faculty of Primary Education, National and Kapodistrian University of AthensAthens, Greece; ^3^Department of Psychology, University of CreteRethymno, Greece; ^4^National Center for Assessment in Higher EducationRiyadh, Saudi Arabia; ^5^College of Education, Taibah UniversityMedina, Saudi Arabia

**Keywords:** non-attempted items, dually attempted items, response styles, guessing, carelessness, differential distractor functioning, IRT, 4-PL

## Abstract

The purpose of the present study was to relate response strategy with person ability estimates. Two behavioral strategies were examined: (a) the strategy to skip items in order to save time on timed tests, and, (b) the strategy to select two responses on an item, with the hope that one of them may be considered correct. Participants were 4,422 individuals who were administered a standardized achievement measure related to math, biology, chemistry, and physics. In the present evaluation, only the physics subscale was employed. Two analyses were conducted: (a) a person-based one to identify differences between groups and potential correlates of those differences, and, (b) a measure-based analysis in order to identify the parts of the measure that were responsible for potential group differentiation. For (a) person abilities the 2-PL model was employed and later the 3-PL and 4-PL models in order to estimate upper and lower asymptotes of person abilities. For (b) differential item functioning, differential test functioning, and differential distractor functioning were investigated. Results indicated that there were significant differences between groups with completers having the highest ability compared to both non-attempters and dual responders. There were no significant differences between no-attempters and dual responders. The present findings have implications for response strategy efficacy and measure evaluation, revision, and construction.

## Introduction

Undoubtedly, being successful on high stakes testing is one of the most important outcomes in one’s young life as consequences involve success, acceptance and positive future outcomes, with opposite effects from failing that testing. For that reason, the unique attributes and characteristics a person brings to a testing situation (such as attitudes and motivation) which likely translate into approaches to test taking are very important for subsequent success or failure. Those characteristics generally belong to individuals’ response styles and are described in detail below. With the term response style/strategy we refer to an individual’s tendency to respond systematically to items regardless of their content (Baumgartner and Steenkamp, 2001). Researchers also agree that an item response is composed of two sources of variance: a true and an error variance (Smith, 2011). Response styles (RS) are considered as sources of systematic error variance, and may become a serious threat for the validity of the scale, since previous research has shown that they can distort tests’ results (Van Vaerenbergh and Thomas, 2012; Sideridis et al., 2014) in various ways. Response styles could affect univariate distributions (e.g., means, variances, etc.) and as a result, could distort results from comparative tests such as *t*-tests or *F*-tests (Cheung and Rensvold, 2000). Response styles could also affect multivariate distributions (e.g., the magnitude of a correlation coefficient between two variables). Since many statistical techniques, such as Cronbach’s alpha, regression analysis, factor analysis, and structural equation modeling, rely on correlations between variables, studies examining such relationships without controlling for RS might yield misleading results (Reynolds and Smith, 2010).

There are several different types of response styles, with the most cited examples being the acquiescence or disacquiescence response style (i.e., the tendency to agree or disagree to an item regardless of content), the mid-point or the extreme response style (i.e., the tendency to give either the middle or the extreme response category), and the socially desirable response style (i.e., the tendency to answer questions in a socially acceptable manner). Less studied but equally important examples, include the mild response style, the net acquiescence response style, the response range, and the non-contingent response style (Van Vaerenbergh and Thomas, 2012).

All the above examples refer to Likert-type items (He and van de Vijver, 2012). There are, however, other types of response styles or response strategies that are presented when dichotomous or multiple-choice items are utilized. Among them, the most common involve guessing on items that are not known and the selection of a subsample of items in order to allocate all resources to items that maximize the probability of success. Thus, individuals may attempt all items (in case there is no penalty for erroneous responding and the measure is not timed) or choose to select items that appear closer to the person’s ability levels. The latter category of respondents is known as non-attempters, since they prefer not to attempt all items of a test, but rather to focus on items that suit their ability levels (Clemens et al., 2015). Other individuals may also choose to select two options with the hope that a rater may be positive toward any one of the responses rather than discarding the responses overall. This response style is known as dual responding (Lepper et al., 2005).

Investigations of response styles have mainly been concerned with the invalidation of scores when method biases residing on person response patterns, such as extreme responding or acquiescence (van Herk et al., 2004) are operative. However, although several studies have examine the role that response styles have on Likert-type items’ attributes (e.g., Baumgartner and Steenkamp, 2001; Weijters, 2006; Weijters et al., 2010a; Van Vaerenbergh and Thomas, 2012) very few studies have examined the role that response strategies play (such as non-attempting all items or dual responding) on both the person and the measure when dichotomous items are involved. In one of these studies, Clemens et al. (2015) examined the hypothesis that if non-attempting an item represents a conscious effort to avoid cognitive source depletion and properly allocate cognitive resources to person-level material, then it may be considered an adaptive strategy. They found that when only complete data were used to estimate total scores (non-attempted items were not considered as incorrect responses) there was a significant increase in performance of 9% points, but only for the low achieving group. This scoring approach was not associated with improved performance for both the middle and the high ability groups.

Clemens et al. (2015) further found that individuals who did not attempt all items had significantly lower performance on a reading comprehension task. However, what is not known is whether that lower performance is a function of true ability, non-exposure to item content, or deficits on prerequisite skills (e.g., lack of fluency, poor vocabulary, etc.).

Another type of response that, to our knowledge, has not been investigated in the past, relates to concurrently responding to two options of an item. Such a response strategy may reflect wishful thinking in hoping that a rater may take one of either options as being correct (thus doubling their chances of success). Another possible explanation is inattention and carelessness to properly follow instructions. However, in most cases dual responses are considered incorrect. Thus, this response style likely represents a maladaptive strategy that is likely related to frustration from facing difficult items, low motivation, and self-regulation failure (Lepper et al., 2005).

The purpose of the present study was to evaluate the effects of response strategy on student’s ability estimates using two behavioral strategies: (a) the strategy to skip items, thus, not completing all items, and, (b) the strategy to over select responses, that is, select two responses on a single item, in relation to individuals who complete the full measure. In an effort to identify such effects and in light of the limitations of previous methodologies (Baumgartner and Steenkamp, 2001; Harzing, 2006), the Item Response Theory (IRT) model is presented as the most applicable model for that research problem (Eid and Rauber, 2000; Gollwitzer et al., 2005). For example, among the explanatory examined factors are the pseudo-guessing and pseudo-carelessness parameters as per the 3-PL and 4-PL, IRT models, respectively. Provided that potential aberrant responding due to carelessness or guessing essentially invalidates measures of ability, the use of the 3-PL and 4-PL models will be implemented as a means to improve measurement efficiency and reduce the possible underestimation of person abilities (Yen et al., 2012). Below there is a detailed description of all three models, and how each one may contribute to our understanding of students’ responding on high stakes testing.

For evaluating differences between groups of responders, four models using contemporary psychometrics are available based on the number of parameters modeled, termed 1 parameter logistic (PL) model, 2-PL, 3-PL, and 4-PL. Furthermore, the latent ability score can be regressed on a grouping variable indicating different response strategies (Bond and Fox, 2001). The 1-PL model (almost equivalent to the Rasch model, Rasch, 1980) will not be implemented herein and thus, is not described. The two-parameter model (Birnbaum, 1968) posits that the probability of correct responding *i* on item *u* for person *j* is given by the expression (Embretson and Reise, 2000; Waller and Reise, 2009):

(1) $$P_{\text{2PL}}(u_{\text{ij}}=1|\;\theta_{\text{i}},\;\alpha_{\text{j}},b_{\text{j}})=\frac{1}{1+e^{\left[-D_{\text{aj}}(\theta_{\text{n}}-B_{\text{i}})\right]}}$$

**FIGURE 1:**
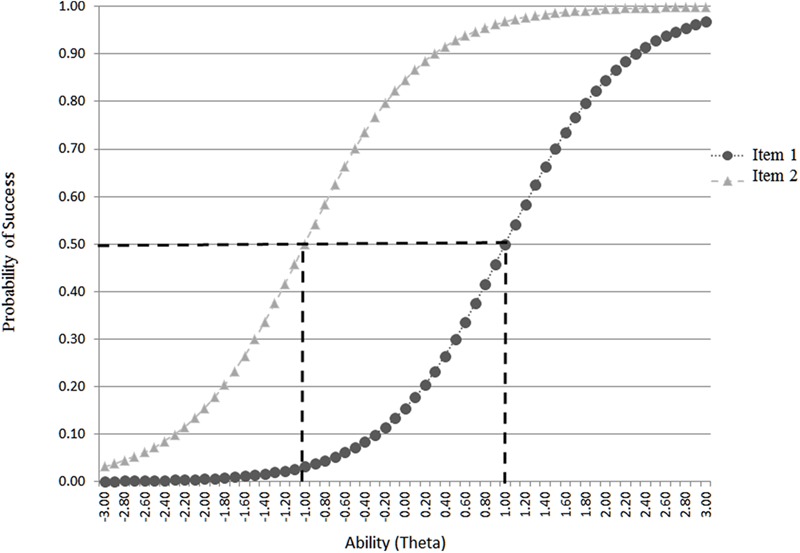
**Example of two item characteristic curve (ICC) curves at -1 and +1 logits of ability**.

With that probability of correct responding *i* being a function of person’s ability 𝜃 and item’s difficulty level *b*. The term *e* = 2.71828 reflects the Euler number and *D* = 1.702 is used to place the item on the normal ogive metric (Wright and Stone, 1979; Wright and Masters, 1982; Crocker and Algina, 1986). The parameter α estimates the degree to which an item discriminates between various levels on the latent trait with steeper slopes associated with greater discrimination and the opposite. Graphically speaking, the relationship between a person’s ability and the difficulty of the item is described by the item characteristic curve (ICC) with item difficulties being located on the horizontal axis and the probability of success on the vertical axis. Thus, the more the curve is to the left, the easier the item is, and the opposite (Baker and Kim, 2004). **Figure 1** shows two hypothetical curves, item 1 associated with below average ability (-1 logit) and item 2 requiring above average ability (+1 logit) to be successful 50% of the time. In order to answer the question with regard to the presence of ‘guessing’ and ‘carelessness’ behaviors, the 3-PL and 4-PL models were employed, respectively (Reise and Waller, 2003; San Martin et al., 2006). The 3PL model estimates item difficulty, item discrimination, and the guessing factor ‘*c*,’ that represents the probability that examinees are successful on an item, for which they do not possess the necessary ability levels (termed pseudo-guessing). With the 3-PL the ‘*c*’ parameter was employed as a proxy to *guessing*, provided that actual guessing was not empirically measured. The 3-PL model is parameterized as follows:

(2) $$P_{3\text{pl}}(u_{\text{ij}}=1|\;\theta_{\text{i}},\;\alpha_{\text{j}},b_{\text{j}},c_{\text{j}})=c_{\text{j}}+(1-c_{\text{j}})\frac{1}{1+e^{\left[-D_{\text{aj}}(\theta_{\text{n}}-B_{\text{i}})\right]}}$$

The 3-PL adds the pseudo-guessing parameter *c*_j_, to assess the magnitude of correct responding for individuals with infinitely low ability levels (Waller and Reise, 2009). The guessing or ‘*c*’ parameter originated in Birnbaum’s (1968) measurement work in an effort to adjust the item response function (IRF, i.e., the logistic curve that describes item level difficulty and discrimination) for very low proficiency individuals who should have a performance of approximately zero but end up having higher than zero performance due to merely random chance in multiple choice questions (i.e., lucky guesses, see Lord, 1974). Liao et al. (2012) demonstrated that even for very low ability individuals the probability of correct response hardly ever approaches zero, thus, advocating in favor of the 3-PL model. Provided there is no objective measurement of guessing, however, Hambleton et al. (1991) properly defined the term as “pseudo-guessing” and this term will be implemented in the present study as well. Estimation of this pseudo-guessing parameter has been justified on the grounds that, particularly for multiple-choice items, guessing will be associated with the provision of credit (partial knowledge) to individuals who do not possess it (Kurz, 1999; Lau et al., 2011) with empirical studies confirming this finding (Betts et al., 2009). The measurement of this pseudo-guessing parameter is particularly relevant and informative in the present study. Provided that individuals who do not attempt all items (e.g., due to response mortality, Clemens et al., 2015) may be of lower proficiency, it is expected that this group would have *lower* values on the ‘*c*’ pseudo-guessing parameter for the following reason. Due to lacking ability, these individuals would have fewer *lucky guesses* as the probability of guessing correctly among four erroneous distractors (which cannot be otherwise eliminated), for which no prior knowledge exist, would be lower compared to higher ability individuals (such as those who complete the measure), who due to higher ability may easily eliminate one or two erroneous distractors and then would guess among one or two possible options (Clemens et al., 2015). In that case, chances for a lucky guess are much higher compared to having to guess among four distractors, for which knowledge to eliminate any one of them is non-existent (Swist, 2015). This prediction was tested in the present study.

Controlling for the lower asymptote accounts for guessing but it is also imperative to account for individual differences in another possible aberrant behavior, that of carelessness (Linacre, 2004). This problem has been posited by Rulison and Loken, (2009) in that early misses severely underestimate person abilities (see also Wen-Wei et al., 2012). Thus, the four-parameter model has been proposed to account for those influences (Barton and Lord, 1981) by allowing the upper asymptote to vary freely across individuals. In simple terms, the 4th parameter estimates the likelihood that high ability individuals miss easy items and adjust person ability scores accordingly, without severely penalizing them. This phenomenon was conceived as reflecting careless errors and, thus, this fourth parameter was used as a proxy to carelessness behaviors. The 4-PL model is parameterized as follows with the addition of the 4th parameter *d*:

(3) $$P_{\text{4PL}}(u_{\text{ij}}=1|\;\theta_{\text{i}},\;\alpha_{\text{j}},b_{\text{j}},c_{\text{j}},d)=c_{\text{j}}+(d-c_{\text{j}})\frac{1}{1+e^{\left[-D_{\text{aj}}(\theta_{\text{n}}-B_{\text{i}})\right]}}$$

For evaluating item level behaviors that could potentially explain group differences a differential distractor functioning analysis (DDF) was conducted to evaluate the behavior of distractors following an omnibus differential item functioning (DIF) test (Raju et al., 1995). For the DIF analysis the M-H procedure was employed (Camilli, 1993) as following:

(4) $$\chi^{\text{2}}=\sum_{i=1}^{L}\frac{D_{\text{i}}^{\text{2}}}{SE_{\text{i}}^{\text{2}}}-\left((\sum_{i=1}^{L}\frac{D_{\text{i}}}{SE_{\text{i}}^{\text{2}}})/\sum_{i=1}^{L}\frac{1}{SE_{\text{i}}^{\text{2}}}\right)$$

with *L* being the items, *D*_i_ the item difficulties (severity levels) of the items *L*, and SE the standard errors of the item difficulties. The chi-square tests the hypothesis that item difficulties for all items *L* are equivalent across all groups. A non-significant test is indicative of DIF absence as it suggests that item difficulties are uniform across groups. The DDF analysis followed the lead of Penfield (unpublished) and evaluates differential option endorsability using the Mantel–Haenszel log-odds ratio (LOR, Mantel and Haenszel, 1959; Camilli and Shepard, 1994). The respective estimate of standard error implemented here was introduced by Robins et al. (1986) and the division of LOR from its SE results in a *Z*-statistic, which is evaluated using a cutoff value of 2.0 units for sample sizes equal to or greater than *n* = 100 cases. The ‘*d*’ or carelessness parameter originated with the work of Barton and Lord (1981) and reflects an upper asymptote that does not lead to 100% performance for high achieving individuals; that is, high achievers fail items that are at their level of ability due to, for example, inattention (Maniaci and Rogge, 2014), stress or carelessness (Barton and Lord, 1981), fatigue or lack of motivation (Huang et al., 2012; Sideridis et al., 2014), insufficient effort (Huang et al., 2012), creative responding (Karabatsos, 2003), or inability to process reverse-worded items (Woods, 2006), among other reasons. Importantly, the presence of careless responding would bias item difficulty parameters negatively, in that the items would appear more difficult than they actually are. Those effects may be particularly more pronounced for speeded tests for which time pressure may lead to careless mistakes (Mroch et al., 2005; Boughton and Yamamoto, 2007; van der Linden, 2007). Thus, statistically speaking the fixed upper asymptote as per the 2-PL, or 3-PL models was left free to vary in the case of the 4-PL. The 4-PL model has recently received increased attention, due to new computationally efficient methods and proof that it assesses more efficiently and with more precision and less error the lower asymptote or pseudo-guessing parameter (Loken and Rulison, 2010). Furthermore the model provides more robust estimates of ability as aberrant responses are down-played having less of an impact on person ability estimates (Magis, 2013). Earlier criticisms of the 4PL model can be traced to the works of Rupp (2003) and Linacre (2004) when efficient estimation methods and software were less accessible. Estimation of the efficacy of the lower asymptote as a means to improve measurement has led to equivocal results, at times favoring (Rulison and Loken, 2009; Loken and Rulison, 2010; Yen et al., 2012) or devaluing its use (Barton and Lord, 1981; San Martín et al., 2015). Despite receiving a lot of criticism as a parameter, however, (Hambleton and Swaminathan, 1985), the fact that careless misses would erroneously lead to a conclusion that an item appears more difficult than what actually is, justified its use (Raiche et al., 2013).

Ancillary to the above measures of pseudo-guessing and pseudo-carelessness, is a set of analyses that aimed at elaborating why guessing may have occurred (under the present correlational design). Thus, a series of distractor analyses were conducted to test the hypothesis that low ability individuals (as likely are those who do not attempt all items) may be attracted in higher rates by erroneous distractors compared to individuals of high ability (as are those who complete all items) (Rogers and Bateson, 1991). Thus, a series of DDF analyses were conducted to examine differential preference to distractors, which may support variability in the estimates of the ‘*c*’ and ‘*d*’ parameters as per the 3PL and 4PL models, respectively. Statistically speaking, a M-H statistic will compare differential responses to distractors between individuals who completed all items and those who (a) did not attempt all items, or (b) provided two responses to any one option. Thus, the over-selection of erroneous distractors will be verified. Visually speaking, by plotting both the correct option and the distractors one will be able to identify percentages of individuals and the respective levels of ability for which a distractor may be the preferred option, over the correct response. This process was described nicely by DiBattista and Kurzawa (2011, p. 1000) who stated that: “An effective distractor will look plausible to less knowledgeable students and lure them away from the keyed option, but it will not entice students who are well-informed about the topic under consideration.” The DDF analyses were run across all items for comparing the reference group (completers) to the two competing groups (non-attempters and dual responders) and the visual analyses involves the behavior of the distractors using two characteristic items.

Thus, the present study will test differences in ability between individuals who complete all items of an ability measure, those who leave items unanswered, and those who respond dually to items with the goal of understanding potential differences in ability as a function of pseudo-carelessness, pseudo-guessing and item-level properties (i.e., quality of distractors). Specifically, the following research questions were addressed in the present study:

RQ1. Are there differences in ability between individuals who complete all items (completers), and those who either over-select items (dual responders) or skip items (non-attempters)?
RQ2. How do the three response strategy groups (completers vs. dual responders vs. non-attempters) vary across different ability levels (low-medium-high)?
RQ3. Can the differential performance between different response strategy groups be explained by differences in pseudo-guessing and pseudo-carelessness?
RQ4. Can differences between response strategy groups be explained by differential preference to incorrect distractors?

## Materials and Methods

### Participants and Procedures

Participants were 4,422 individuals who were administered a standardized measure of achievement related to physics, biology, math, and chemistry. There were 1870 females (42.3%) and 2,549 males (57.6%), the typical distribution of examinees in Saudi Arabia. Data on gender were missing from three individuals (0.001%). The participants were provided with a standard set of instructions using a power-point presentation. They were allowed to wear or carry a watch. Among those tested, 2,211 had completed all items and became the reference group when comparing individuals exhibiting variable response styles. This group was selected at random from a larger national sample of 63,349 participants. Another group of participants (*n* = 2,211) was bifurcated onto two groups (a) 1,030 individuals who left unanswered 1 or more items (ranging between 1 and 16 items), and, (b) 1,181 individuals who had marked more than one option at any one item (with the number of items displaying dual responding ranging between 1 and 17). Provided that 62.8% of the participants had selected two responses on a single item only, this group was combined to a group formed of individuals employing this strategy in more than one, items. Results indicated miniscule differences between the two groups with Cohen’s *d* being 0.27 indicating a small effect size. Non-attempters could potentially be bifurcated onto a subgroup that fails to attempt the last few items (termed response mortality) due to running out of time or individual’s perceptions that continuous effort would not lead to meaningful consequences (Clemens et al., 2015). Inspection of the presence of such a subgroup suggested that there were only 19 individuals (representing 0.018% of the total sample) who displayed that pattern of responding. Consequently, these individuals could not comprise a group for further analyses. Based on standard scoring procedures of the measure, all empty cells (non-attempted), and dual response cells were marked as incorrect responses, and this practice was followed in the present study as well. Specifically, for dual responders a value of zero was assigned regardless of whether one of the two responses circled was correct. Last, as a thoughtful reviewer suggested individuals could present both response styles (non-attempts and dual responses). A group of 62 individuals presented themselves with that pattern of responding but were excluded from further analysis due to their small sample size, and consequently the low levels of power associated with fitting an IRT model to that group (Hollman et al., 2003).

Three ability groups were formed, independently of response strategy, using 33 and 67% percentile values as cutoff points to define low-medium-high ability individuals based on summed performance on the Physics scale. This grouping variable was tabulated with the response style grouping variable to test the hypothesis that different response style groups are associated with differential ability levels.

### Measure

*The Standardized Achievement Admission Test* (SAAT; National Center for Assessment in Higher Education) was employed and specifically the Physics subscale. The total measure involves four subscales (biology, chemistry, physics, and mathematics) involving 130 items. The level of the test corresponds to 3rd year curriculum in Saudi Arabia high schools. The Physics subscale includes 20 items and is timed with all items completed in a maximum of 30 min. Sample content involves properties of matter, elasticity, mechanics of fluid and atmospheric pressure (for two sample items, see Appendix A). Reliability of the measure was verified using composite reliability omega (Raykov, 2006) and was found to be 0.723 whereas maximal reliability of the weighted composite score was equal to 0.745 (Geldhof et al., 2014). Finally, it should be noted, that the study was conducted as part of a National Examination in Saudi Arabia. All ethical procedures have been monitored closely by the examination body (i.e., National Center for Assessment in Higher Education - NCA).

### Data Analysis

Provided that the IRT models were described above, this section contains information on the analysis of distractors when DIF is initially observed. A DDF analysis is ancillary and complementary to DIF and targets at decoding and understanding the DIF findings through examining how each of the response options contributes to measurement and whether those options are invariant across groups (Dorans et al., 1992; Penfield, unpublished). Significant DIF is a desirable but not necessary condition to testing the differential behavior of distractors. When DIF is significant, the DDF analysis shows which distractors bias the correct response; that is, differential achievement evidenced by the DIF is interpreted via analyzing the distractors. A DDF analysis, however, does not require a significant DIF effect; a distractor may be differentially attractive to one group of people compared to another, without affecting ability on the item overall. In other words, DDF may be evident when two groups select two different erroneous options on an item; thus, they both are unsuccessful but presented themselves with differential selections of item options.

As previously mentioned, Penfield (2008) proposed the odds ratio (OR) method as a means for estimating DIF and DDF effects. This method is modeled under both, the nominal response model (Bock, 1972) and the multiple-choice model (Thissen and Steinberg, 1986). This model in fact extends the Mantel–Haenszel (MH) method (Mantel and Haenszel, 1959), later altered by Holland and Thayer (1988) for analyzing dichotomously scored items. For item *i*, the MH common odds ratio is computed using the following expression:

(5) $${\hat{\alpha }}_{\text{MHi}}=\frac{\sum_{s=1}^{s}R_{1\text{s}}F_{0\text{s}}/n_{\text{s}}}{\sum_{s=1}^{s}R_{0\text{s}}F_{1\text{s}}/n_{\text{s}}}$$

When the odds ratio is converted to a LOR by taking the natural log, the value becomes a signed index. This signed index is referred to as 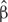
_MH_ and is calculated by 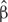
_MH_ = ln(â_MH_), where a positive value indicates DIF in favor of the reference group, and a negative value indicates DIF in favor of the focal group. A value equal to zero indicates no DIF effect.

## Results

### Prerequisite Analyses

Prior to answering the focal research questions, it was important to establish that the measurement models (2-PL through 4-PL IRT) fit the data well. Thus, evidence in favor of the 2-PL to 4-PL models is presented herein as meaningful conclusions regarding group differences could not be drawn in the absence of proper psychometric properties of the Physics measure. Those results are shown in **Table 1**. Model fit was evaluated using the omnibus Chi-square discrepancy test with a significant finding suggesting non-negligible misfit. Additional evidence, particularly useful for model comparison was provided by information indices (AIC, BIC, and CAIC). As **Table 1** shows, both 3-PL and 4-PL models fit the data well with the modeling of the upper asymptote being adaptive (i.e., it was associated with better model fit compared to the 3-PL model). Thus, adjusting the lower asymptote for guessing and the upper asymptote for carelessness was particularly relevant and informative using the present sample. The 2-PL model appeared to misfit; however, in order to rule out the hypothesis that the observed misfit was not a function of excessive levels of power, the model was re-run using a random sample of *n* = 500 participants. Results indicated that the new Chi-square statistic was no longer significant [χ^2^(380) = 86.535, *p* = n.s.]. Thus, all findings corroborated with the idea that the 2-PL model was a good fit to these data.

**Table 1:** Model Fit of Physic Scale as per 2-PL, 3-PL, and 4-PL Models.

Model tested	Chi-square	AIC^†^	CAIC^†^	BIC^†^
2-PL	686.257^∗∗∗^	-73.743	-2894.939	-2514.939
3-PL	281.098	-438.902	-3111.614	-2751.614
4-PL	26.446	-653.554	-3177.782	-2837.782

*^∗∗∗^*p* < 0.001. AIC, Akaike Information Criterion. BIC, Bayesian Information Criterion. ^†^AIC, Akaike Information Criterion; CAIC, Consistent AIC; BIC, Bayesian Information Criterion.*

**R.Q.1: Are there differences in ability between individuals who complete all items (completers), and those who either over-select items (dual responders) or skip items (non-attempters)?**

**FIGURE 2:**
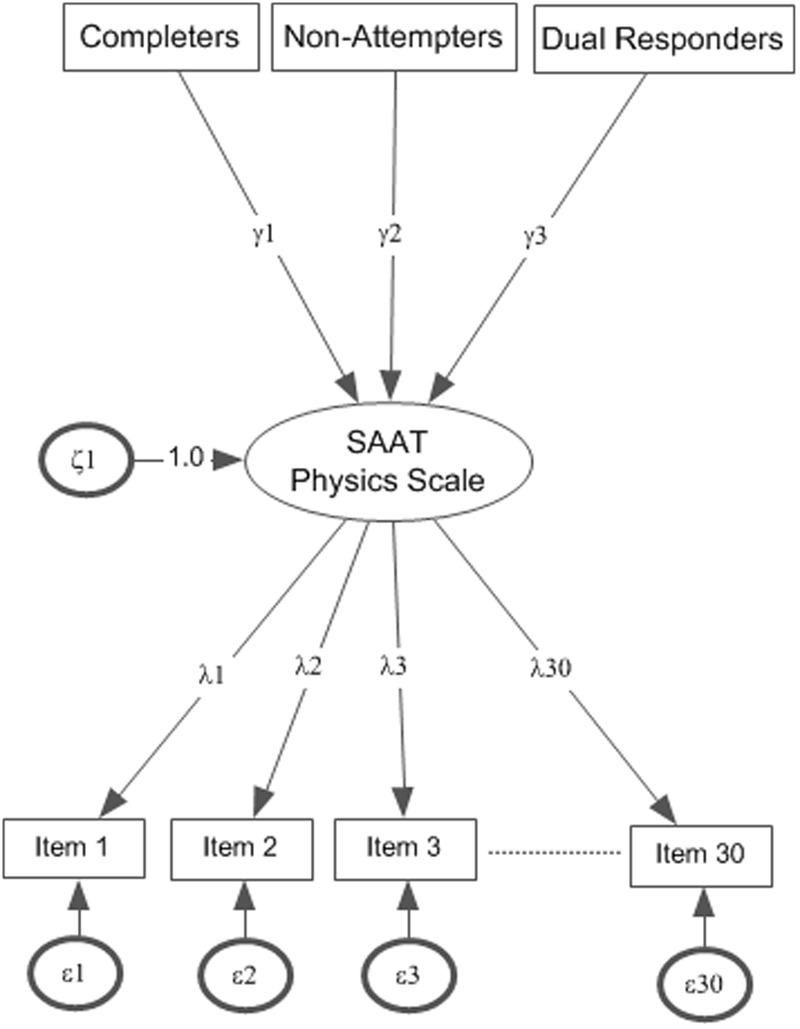
**Mean ability estimates for the three groups on Physics achievement based on the 2-PL model.** Actual point estimates γ_1_–γ_3_ are shown in **Table 1**.

To answer the first research question, the 2-PLmodel was fit to the data simultaneously for the three groups of students and mean ability estimates were assessed in logits (see **Figure 2** for model tested, parameters γ_1_–γ_3_ relate to mean comparisons). Following this equating procedure, as **Table 2** shows, the mean ability levels of the random sample that completed all items was 0.712 logits. Thus, those individuals were of higher than average ability after conditioning for overall ability. For individuals who failed to attempt from few to several items, mean ability levels were at -0.015 logits, suggesting approximately average ability. At similar levels was the ability of individuals who attempted two responses within any one item (i.e., -0.033 logits). The difference between groups was significant [*F*(2,4419) = 236.104, *p* < 0.001] (see estimates in **Table 2**). Using the Tukey *post hoc* test and controlling for the number of comparisons using the *Q* statistic (Pearson and Hartley, 1970), results indicated significant differences between the reference group (who completed all items) and all other groups with the group which completed all items having significantly higher mean ability levels. There were no significant differences between the Non-Attempters and the Dual response groups. Thus, not attempting items and dual response appears to represent two *‘unsuccessful’* response strategies associated overall with lower performance compared to completing all items. The same differences in ability were essentially replicated, although augmented, in favor of the reference group which completed all items when fitting the 3-PL and 4-PL models, respectively, that is after adjusting person abilities for pseudo-guessing and pseudo-carelessness responses. Again, differences between groups were significant (see **Table 2**) with the group who completed all items having significantly higher levels of ability compared to both comparison groups after fitting the 3-PL [*F*(2,4419) = 235.981, *p* < 0.001] and 4-PL models [*F*(2,4419) = 234.632, *p* < 0.001]. No-Attempters and dual response groups were again no different in ability as per the 3-PL and 4-PL models.

**Table 2:** Mean estimates (Thetas) per group of responders as Per 2-PL, 3-PL, and 4-PL models.

	Random sample		Dual
Model tested	completers	Non-attempters	responders
2-PL IRT^†^	0.712	-0.015	-0.033
3-PL IRT	0.210	-0.579	-0.611
4-PL IRT	0.599	-0.240	-0.263

*The common item equating procedure was followed in the above estimation of person abilities for each of the two models. ^†^2-PL stands for 2-Parameter Logistic Model.*

**R.Q.2. How do the three response strategy groups (completers vs. dual responders vs. non-attempters) vary across different ability levels (low-medium-high)?**

**FIGURE 3:**
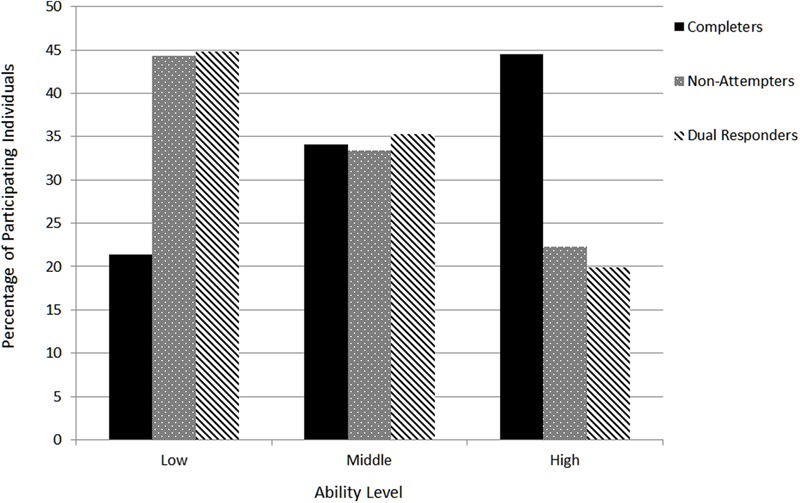
**Relationship between response style groups and three hypothetical levels of achievement (low-medium-high).** Percentages are for each achievement level conditional on response style group and add to 100% for each response style group.

The present research question was answered by cross tabulating response strategy groups (i.e., completers, non-attempters and dual responders) with different ability groups (i.e., low, medium, and high scorers on the Physics subscale). **Figure 3** displays the findings from the cross tabulation with Pearson’s Chi-square value being significant [χ^2^(4) = 367.348, *p* < 0.001]. Pearson’s *r* was equal to -0.267 (*p* < 0.001) suggesting that moving from completers to non-attempters and dual responders was associated negatively with achievement grouping (low-medium-high). As **Figure 3** shows most participants of the reference group (i.e., completers) belonged to the ‘high-ability’ group compared to no-attempters and dual responders who were saliently represented in the ‘low-ability’ group first, followed by the ‘medium’ ability group. Thus, both groups who did not complete all items or involved dual responding appear to be underrepresented in the high ability group, occupying mostly low to middle levels of achievement on the physics measure.

**R.Q.3. Can the differential performance between different response style groups be explained by differences in pseudo-guessing and pseudo-carelessness?**

The purpose of this analysis was to attempt to explain differences in ability between the different response strategy groups given the parameters of pseudo-guessing ‘*c*’ (lower asymptote of the 3PL IRT model) and pseudo-carelessness ‘*d*’ parameter (upper asymptote of the 4PL IRT model). Those analyses were run to address the hypothesis that non-attempters and dual responders may engage in either one of these behaviors during test-taking. Guessing, for example, represents successful attempts to answer items for which adequate levels of knowledge are absent, thus, high scores on guessing represent successful attempts as the individuals guessed the correct response, albeit the fact that person levels of ability cannot justify that [*successful*] performance (Han, 2012). Carelessness, on the other hand reflects misses on items (errors) for which person performance cannot explain (in that adequate levels of performance are present but the person still misses those items). It is important to note here, however, that the causes of aberrant responding (reflected in high values in the lower asymptote and low values in the higher asymptote) cannot be supported from the present design and are only speculative of the processes that likely take place during test-taking.

For guessing the expectation was, given findings from research questions 1 and 2, that individuals who chose not to attempt items or respond twice on an item would have lower ‘*c*’ parameters reflecting a larger number of incidences of *unsuccessful guessing*. This expectation is based on the fact that guessing reflects *successful* choices and such attempts may be more frequently observed in individuals of higher ability compared to low achievers. Particularly for the latter group (which most of the non-attempters and dual responders actually were in the present study), it was expected that low achievement would be associated with unsuccessful guessing, that is an attraction to incorrect distractors (see next research question for evidence to that effect).

**FIGURE 4:**
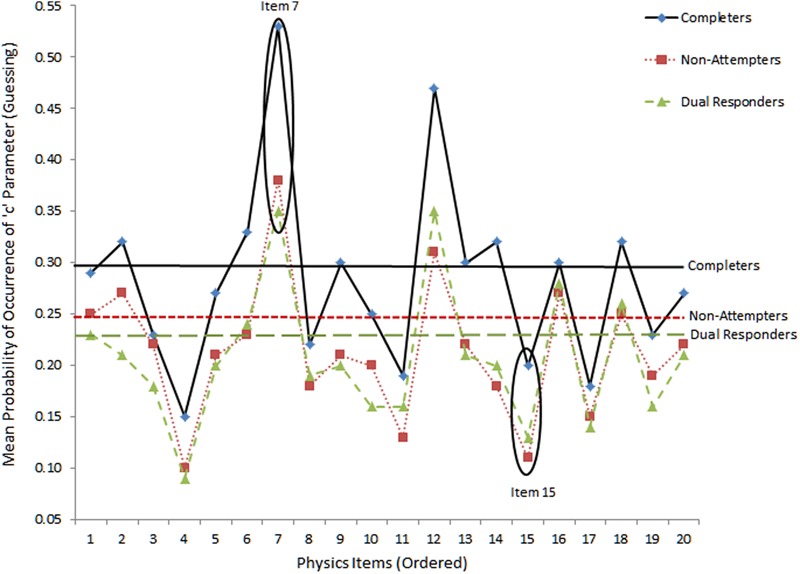
**Mean levels of lower asymptote parameter (i.e., pseudo-guessing) as per 3-PL model.** Horizontal bars reflect mean estimates per group. Absence of guessing would be reflected with values equal to zero.

**FIGURE 5:**
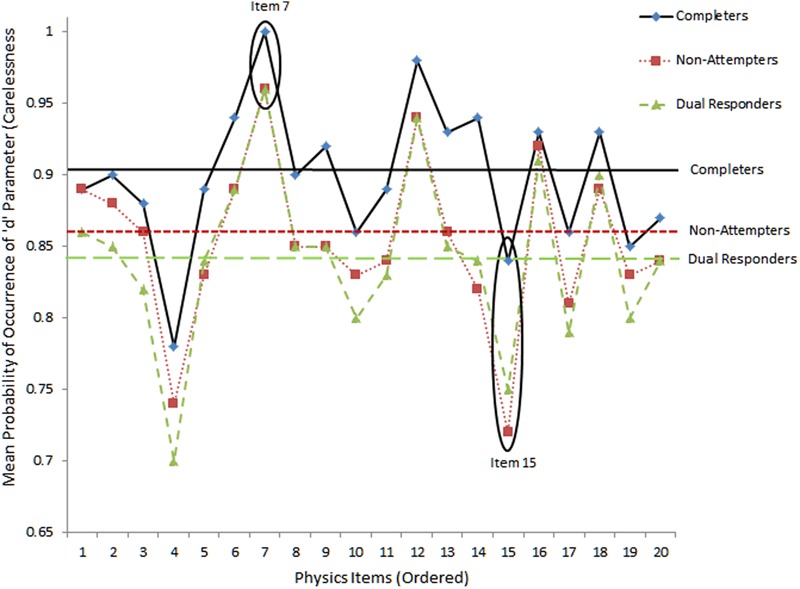
**Mean levels of upper asymptote parameters (i.e., carelessness) as per 4-PL IRT model.** The horizontal bars reflect mean estimates per group.

For carelessness, the expectation was that those instances are more prevalent to lower ability individuals, such as those who fail to attend properly to instructions (as both dual responders and non-completers likely are). For example, if dual response patterns reflects inattention, and carelessness, the “pseudo-carelessness” statistic may be more elevated for that group compared to others. This relative conclusion may be grounded on the hypothesis that low ability individuals may appear disorganized and may employ ineffective strategies when challenged by test content. **Figures 4** and **5** display item-level data for lower and upper asymptotes, respectively. The absence of guessing and carelessness would be evident with expected values in the lower and upper asymptotes of 0 and 1, respectively. **Figure 4** shows the results on pseudo-guessing with mean levels (shown using horizontal lines) being significantly higher for the reference group of completers compared to both comparison groups [*F*(2,57) = 6.065, *p* < 0.01], using Tukey’s *post hoc* procedure. Note that these comparisons involve mean levels of guessing at the item level, not the person. There were no significant differences in the levels of the pseudo-guessing item parameters between the no-attempters and the dual responding groups. Items 7 and 15 were circled because guessing appeared at high and low levels, respectively, for all groups. Item 7 represented a low difficulty item (i.e., -0.341 logit), thus, high scores on the ‘*c*’ parameter suggests successful guessing. On the other hand, item 15 was one of the most difficulty items on the present physics measure (i.e., 0.796 logits), thus, successful guessing was less probable for all participants (although it appears to be more probable for the more able group of completers most likely due to properly ruling out erroneous distractors).

**Figure 5** shows item and group mean levels of the ‘*d*’ pseudo-carelessness parameter using the 4-PL model. For the absence of carelessness, the expectation is that the probability of occurrence of the d parameter is 1 (i.e., no lower than expected performance due to carelessness). The values on the vertical axis reflect actual values of ‘*d*’ parameters for each item. Items 7 and 15 were circled with the former showing little careless errors (as it is an easy item) and the latter large amounts of carelessness, more so for low ability groups (no-attempters and dual response participants). Differences between groups were significant groups using the omnibus ANOVA test [*F*(2,57) = 5.572, *p* < 0.01]. Using Tukey’s *post hoc* test results pointed to significantly higher levels of pseudo-carelessness for non-attempters and dual responders compared to completers. No significant differences were observed between dual responders and non-attempters.

**R.Q.4. Can differences between response strategy groups be explained by differential preference to incorrect distractors?)**

The previous research question pointed to the direction that differential between groups performance could be attributed to either guessing or carelessness behaviors, via investigating the magnitude of the respective pseudo-guessing and pseudo-carelessness parameters. A thorough analysis at the item level was conducted to evaluate those claims. For example, one hypothesis in the presence of lower levels in the pseudo-guessing parameter for the group who completed all items compared to all other groups was that the former group had higher ability levels (as verified by findings from research questions 1 and 2) and, thus, guesses for completers, when employed, would most likely lead to success through eliminating erroneous distractors, even if knowledge on the correct response was lacking. On the contrary, for low achievers, as the groups of non-attempters and dual responders mostly were, there was a higher likelihood to be attracted by erroneous distractors, due to simple inability to differentiate between various erroneous responses. This hypothesis was tested by use of the DDF analysis following an omnibus significant DIF effect. Evidence in favor of this hypothesis was provided when erroneous distractors were significantly more attractive to the non-attempters and dual responders, compared to completers.

**FIGURE 6:**
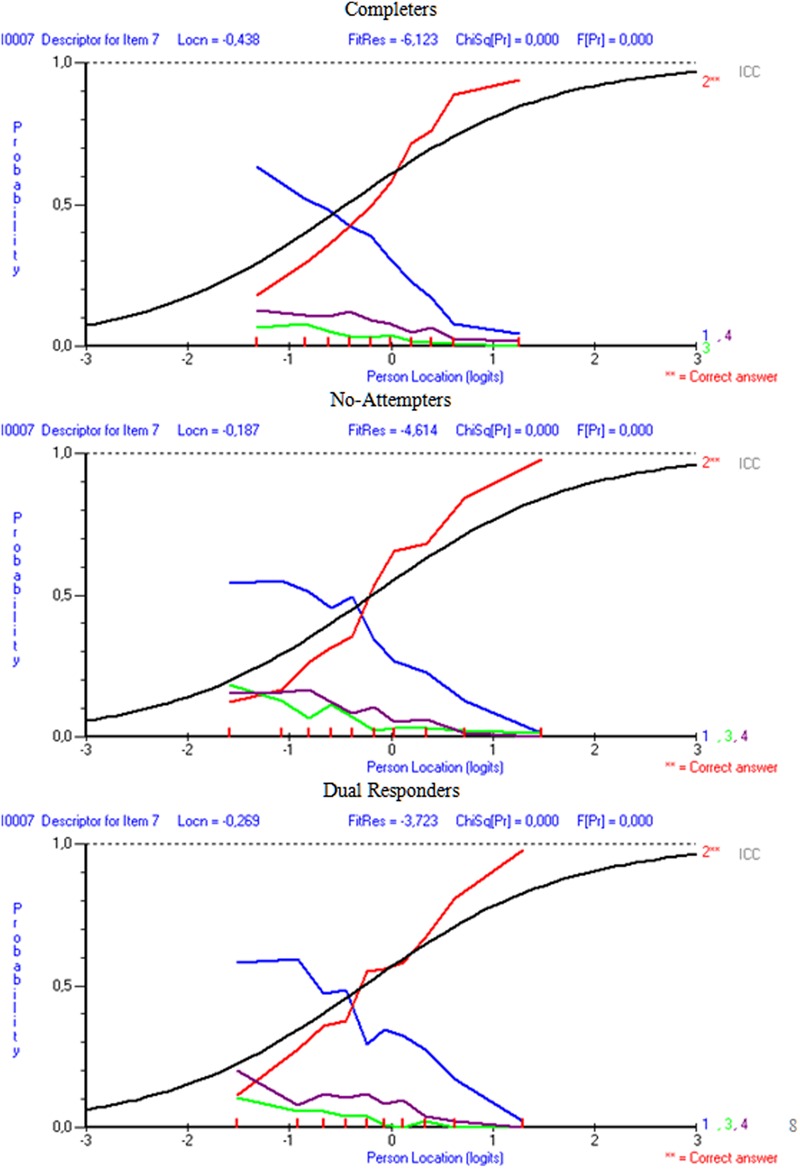
**Distractor behaviors for item 7 of the Physics subscale per response style group**.

**FIGURE 7:**
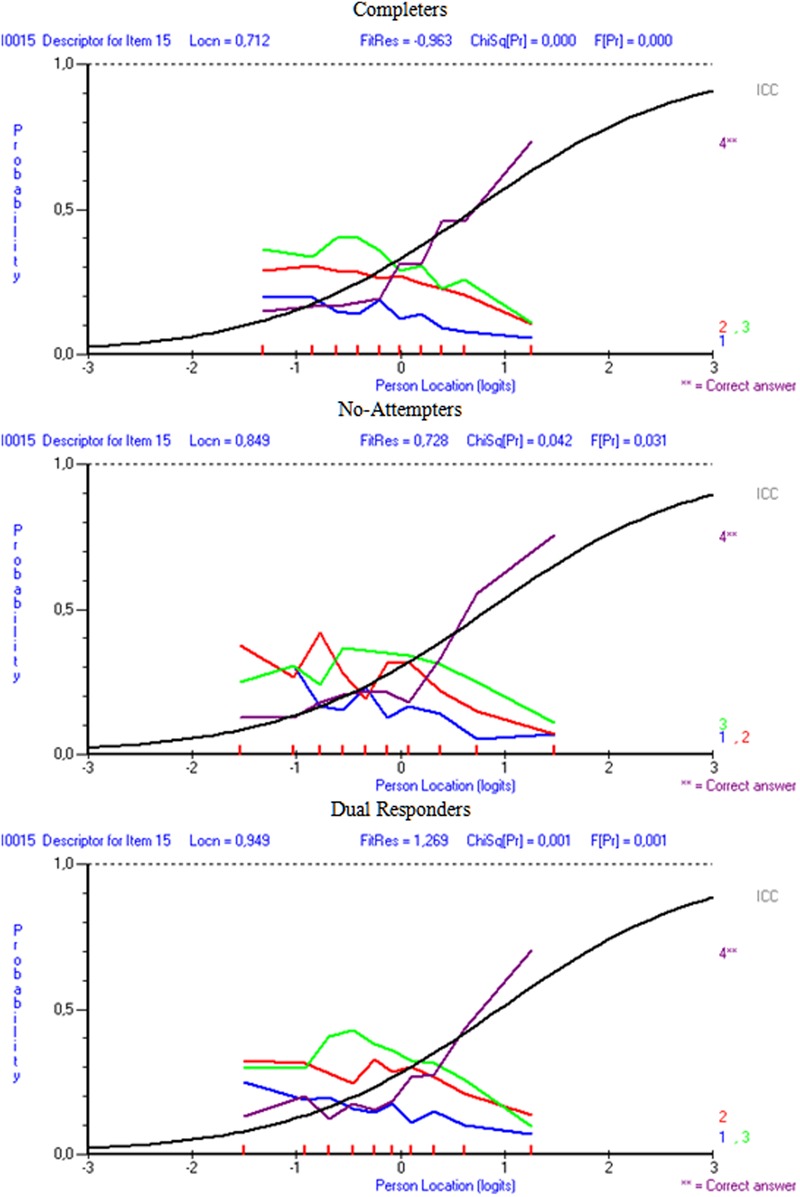
**Distractor behaviors for item 15 of the Physics subscale per response style group**.

Results indicated that in the comparison between completers and non-attempters there were 13 items out of the 20 in which erroneous distractors were disproportionally more attractive to non-attempters compared to completers. Similarly, when comparing dual responders with completers, there were 11 items in which distractors were significantly more attractive in the dual response group compared to the completers, after conditioning for ability. **Figures 6** and **7** display distractor information for items i7 and i15 in order to elaborate on the DDF findings and their relationship to pseudo-guessing and pseudo-carelessness per group of participants. As the item 7 figure shows (**Figure 6**) distractor 1 was significantly more attractive to non-attempters (Option 1_LOR_ = 0.325, *Z* = 3.250, *p* < 0.01), and dual responders (Option 1_LOR_ = 0.313, *Z* = 3.324, *p* < 0.01) in comparison to the reference group (completers), based on DDF analysis and the LOR test (Penfield, 2011; Penfield, unpublished). For dual responders more than 50% of the participants who were of sub-average ability (below zero logits) selected this option, compared to approximately 50% for the non-attempters and a negative trend going below 50% for the completers. Thus, both groups were heavily distracted by this option, which lead to unsuccessful guessing (i.e., lower levels on ‘*c*,’ the pseudo-guessing parameters). Item 15 (**Figure 7**) shows distractors for all three groups of participants. The results from the DDF analyses suggested that all distractors were disproportionally more attractive to the dual responders compared to completers (Option 1_LOR_ = 0.367, Z = 2.784, *p* < 0.01; Option 2_LOR_ = 0.363, *Z* = 3.302, *p* < 0.01; Option 3_LOR_ = 0.313, *Z* = 2.935, *p* < 0.01) as again this low achieving group seems to be significantly more attracted by those erroneous options. The respective findings for the non-attempters compared to completers were significant for distractor 1 (Option 1_LOR_ = 0.336, Z = 2.299, *p* < 0.01) using a two-tailed test, which is at higher levels for dual responders of lower ability compared to completers of lower ability, but also distractors 2 and 3 using a one-tailed test suggesting the presence of a trend (Option 2_LOR_ = 0.221, *Z* = 1.757, *p* < 0.05 one-tailed; Option 3_LOR_ = 0.214, *Z* = 1.781, *p* < 0.05 one-tailed). These findings provide support to the hypothesis that individuals of low ability, as non-attempters and dual responders were, display unsuccessful guessing (lower levels of ‘*c*’ parameters), likely because they are attracted significantly more by erroneous distractors compared to higher ability individuals.

When looking at the relationship between distractor behavior and pseudo-carelessness, item 7, being an easy item, was associated with low carelessness as individuals having more than -0.7 logits of ability were able to answer it correctly 50% of the time (see **Figure 5**), Item 15, however, was one of the most difficult items, and, some of the few individuals of higher than the required item 15 ability that belonged to non-attempters and dual responders seemed to have failed it (i.e., they had high values on the ‘*d*’ parameter, see **Figure 5**). This finding, however, is only correlationally linked to the hypothesis of altered emotional states and unsuccessful self-regulation and should be viewed with caution.

## Discussion

The purpose of the present study was to evaluate the effects of response strategy on student’s ability estimates using two behavioral strategies: (a) the strategy to skip items in order to save time on timed tests (non-attempters), and, (b) the strategy to select two responses on an item, with the hope that one of them may be considered correct (dual responders). The reference point was a group of individuals who completed all items (completers), selected at random from a population of high school students who took on a specific national entrance examination for entry in higher education.

The most important finding was that non-attempters and dual responders represented the lowest ability groups. Contrary to expectations, non-attempting an item was not an adaptive strategy in which an individual skipped unknown items with the purpose of focusing all cognitive resources to items for which some knowledge was present. Non-attempted items were not given a chance of being correct (not even in the form of a guess) thus, likelihood of success, even due to chance, lowered. Similarly, dual responding, albeit explicitly stated as a negative response style (in that all such responses would be penalized) still elicited a significant amount of endorsement. Apparently, there was some hope that one of two responses could eventually be considered correct. The authors consistently returned to the procedures involved and directions during test taking but it was verified that all procedures were standardized using a PowerPoint presentation. Thus, there was little doubt that dual responding was actually due to ambiguous instructions prior to the test.

Another important finding was that non-attempters and dual responders likely emitted large amounts of unsuccessful guessing, as the pseudo-guessing parameters (which reflect successful guessing) were significantly lower for them compared to completers. Furthermore, the present study attempted to elucidate the relationship between guessing and low achievement through investigating the behavior of the distractors. Results pointed to the presence of significantly higher preference for erroneous distractors of the two response style groups (non-attempters or dual responders) compared to the reference group (completers), even after conditioning for ability (via the DDF analysis). Furthermore, a visual analysis of the distractors confirmed that unsuccessful guessing occurred for low achieving individuals (as the two response style groups mostly were), as high levels of endorsability of erroneous distractors were evident for individuals having below average levels of ability (see item 7, **Figure 6**). Thus, the present findings confirmed the hypothesis that in the presence of low achievement, unsuccessful guessing likely takes place. These individuals have trouble disregarding erroneous distractors in relation to the correct response. It has been suggested that non-attempts could buffer the negative affect of being disappointed and frustrated from attempting difficult items (Clemens et al., 2015). Thus, this strategy has been recommended in order to preserve optimal levels of motivation and avoid the vicious cycle of helplessness, hopelessness, and, eventually effort withdrawal (Swendsen, 1998). However, the present study did not provide support in favor of this self-preservation hypothesis as the design (field testing) did not allow for additional measurements and experimental manipulations.

A third important finding related to the fact that careless responding, via the pseudo-carelessness parameter, observed in higher rates in the dual responders, compared to both non-attempters and completers. This finding confirms the hypothesis that individuals who fail to adhere to explicit instructions about the impending penalty from adopting this behavior, may have been inattentive, impulsive or both. Maniaci and Rogge (2014) defined carelessness as non-compliance with study tasks such as following directions properly. The literature on self-reported measures has confirmed rates of inattention that ranged between 3 and 46% (e.g., Berry et al., 1992; Johnson, 2005; Oppenheimer et al., 2009; Meade and Craig, 2012; Maniaci and Rogge, 2014). Non-attempters also had higher levels on pseudo-carelessness, a finding that agrees with the behavior of that group on pseudo-guessing. That is, since unsuccessful guessing was observed in that group, and the hypothesis that non-attempting items preserves individuals’ resources and focuses them on manageable content was not supported, it looks like non-attempters by failing to focus actually displayed the opposite behavior, that of carelessness. Thus, both unsuccessful guessing and carelessness were observed in higher levels compared to the group which attempted all items. The present study successfully employed the pseudo-carelessness parameter as a proxy to true carelessness and future studies may confirm the role of that parameter in estimating carelessness, compared to previous means (e.g., infrequency scale, Meade and Craig, 2012).

Of great interest was the behavior of the groups on the upper asymptote in that, for example, response mortality may be accountable for the non-attempters. Partial response mortality, that is the general tendency to not respond to items (not just the last ones) may be indicative of altered motivational and emotional states such as the presence of maladaptive motivation, fatigue, negative affectivity, or hopelessness (Emons, 2009; Clemens et al., 2015). Those attributes may be potential explanations for the findings related to the high levels in the ‘*d*’ parameter of pseudo-carelessness for both, non-attempters and dual responders. For example, if altered emotional states are present in non-attempters, the presence of disorganization may be accountable for displaying careless mistakes. On the other hand, dual responders represent a group who chose to respond to an item using two options, despite the fact that explicit directions by examiners clearly pointed to the negative consequences from employing that strategy on test performance. If inattention is one of the causes for this group of dual responders to ignore directions, this lack of concentration will also likely be responsible for careless mistakes.

Furthermore, as a thoughtful reviewer suggested, the linkage between low achievement and, for example, failing to respond to an item may be perceived as a directional one in the present study. This, however, cannot be the case with our correlational design. Thus, it is equally correct to state that individuals who are low achievers will most likely skip items and those who skip items will eventually have lower performance. The picture may be far more complex as variables such as agitation and apprehension from simply being in the testing situation, maladaptive motivational pursuits, personality predispositions, and other less known factors can be the causal indicators in altering the relationship between achievement levels and non-responding or dual responding. As a reviewer stated: “Would a poor response strategy lead to low achievement or would low initial achievement lead to engaging in poor response strategies?”

One can only speculate what the causes were of inattention and the ability to focus on the relevant information. Cognitive science has provided some support to the fact that when individuals are negatively motivated (as individuals who face insurmountable in difficulty item) different areas on the brain are activated (the ventral striatum, fusiform gyrus, left dorsolateral prefrontal cortex (DLPFC) and ventromedial prefrontal cortex), which eventually result in no significant gains on task performance (Reckless et al., 2013).

The present study has several implications. At the test level, it will be important to evaluate the efficacy of each option/distractor and their contribution to construct validity. One aspect of the distractors tested earlier was in relation to the number of available distractors with results suggesting that the number of options was unrelated to success (Bruno and Rutherford, 2010). Another implication relates to how non-attempted items will be treated (Enders, 2001, 2006; Enders and Peugh, 2004), either as missing scores or zeros with the former having implications on the type of data missingness (e.g., MCAR, etc.). At the person level it is important to decide on the number of non-attempts that still constitute a valid response pattern and both the use and interpretation of scores derived from non-attempters (Eason et al., 2012). If fatigue is implicated (Arvey et al., 1990; Chan et al., 1997; Ackerman and Kanfer, 2009), then one option may be to cut the test into multiple, manageable, administration times (DiCerbo et al., 2004). Another implication relates to the relationship between non-attempting items and timed tests, particularly for individuals with disabilities (Lewandowski et al., 2013). Should those individuals be provided with accommodations to ensure item content has been comprehended?

The present study is also limited for several reasons. First, response mortality could not be investigated in the non-attempters group as individuals who did not attempt the last few items of the measure could not comprise a group (*n* = 19). Second, inferences about guessing and carelessness are made throughout the manuscript (termed *pseudo*) but it is important to note that those terms are used for estimated statistics and reflect, in the best case scenario proxy estimates of those constructs. A quasi experimental design to evaluate those constructs via self-report or observations would be more appropriate. Third, the findings have implications about participants’ motivation, affect and anxiety, but these constructs were not specifically measured. Fourth, although intended, we were unable to create a combined group presenting both response styles (non-attempts and dual responses) due to again a small sample size (*n* = 59) (Linacre, 1994). Thus, possible explanations of the findings related to effective or ineffective self-regulation are speculative, and, should be viewed under the lenses of the present correlational design; by no means should causal inferences be made.

In the future it will be important to measure traits and states that have been recommended in the literature to predict task engagement and achievement in line with the recommendations of Eid and Rauber (2000) and Bolt and Johnson (2009) to incorporate responses style within the psychometric model (see also Gollwitzer et al., 2005). Achievement goals, emotions, negative affectivity processes and personality need to further be investigated and their role to be tested in self-regulation and achievement (Furnham et al., 2015). This may be done more so with an extension of analytical approaches presented by Moors (2003, 2004), Abad et al. (2009), Weijters et al. (2010b), Gattamorta et al. (2012), and Culpepper (2015). If carelessness and guessing are the actual causes of lower performance (Woods, 2006), specific accommodations such as the use of virtual presence (Ward and Pond, 2015) need to be implemented and new analytical approaches need to be applied to improve the way these phenomena are modeled (Burton, 2001; Unlu, 2006; Glas, 2009).

## Author Contributions

GS: He analyzed the data and wrote part of the Introduction, Results, and Discussion sections. IT: He contributed in reviewing the literature and wrote part of the Introduction Section. He also contributed in the Results and the Discussion sections. KA: He was responsible for the data collection, and wrote part of the Methodology section.

## Conflict of Interest Statement

The authors declare that the research was conducted in the absence of any commercial or financial relationships that could be construed as a potential conflict of interest.

## References

[B1] Abad F. J. Olea J. Ponsoda V. (2009). The multiple-choice model: some solutions for estimation of parameters in the presence of omitted responses. *Appl. Psychol. Meas.* 33 200–221. 10.1177/0146621608320760

[B2] Ackerman P. L. Kanfer R. (2009). Test length and cognitive fatigue: an empirical examination of effects on performance and test-taker reactions. *J. Exp. Psychol. Appl.* 15 163–181. 10.1037/a0015719 19586255

[B3] Arvey R. D. Strickland W. Drauden G. Martin C. (1990). Motivational components of test taking. *Pers. Psychol.* 43 695–716. 10.1111/j.1744-6570.1990.tb00679.x

[B4] Baker F. B. Kim S. (2004). *Item Response Theory: Parameter Estimation Techniques* 2nd Edn New York, NY: Marcel Dekker.

[B5] Barton M. A. Lord F. M. (1981). *An Upper Asymptote for the Three-Parameter Logistic item-Response Model.* Princeton, NJ: Educational Testing Service.

[B6] Baumgartner J. E. M. Steenkamp H. (2001). Response style in marketing research: a cross-national investigation. *J. Mark. Res.* 38 143–156. 10.1509/jmkr.38.2.143.18840

[B7] Berry D. T. R. Wetter M. W. Baer R. A. Larsen L. Clark C. Monroe K. (1992). MMPI-2 random responding indices: validation using a self-report methodology. *Psychol. Assess.* 4 340–345. 10.1037/1040-3590.4.3.340

[B8] Betts L. R. Elder T. J. Hartley J. Trueman M. (2009). Does correction for guessing reduce students’ performance on multiple-choice examinations? Yes? No? Sometimes? *Assess. Eval. High. Educ.* 34 1–15. 10.1080/02602930701773091

[B9] Birnbaum A. (1968). “Some latent trait models and their use in inferring an examinee’s ability,” in *Statistical Theories of Mental Test Scores* eds Lordand F. M. Novick M. R. (Reading, MA: Addison-Wesley).

[B10] Bock R. D. (1972). Estimating item parameters and latent ability when responses are scored in two or more nominal categories. *Psychometrika* 37 29–51. 10.1007/BF02291411

[B11] Bolt D. M. Johnson T. R. (2009). Addressing score bias and differential item functioning due to individual differences in response style. *Appl. Psychol. Meas.* 33 335–352. 10.1177/0146621608329891

[B12] Bond T. G. Fox C. M. (2001). *Applying the Rasch Model* 2nd Edn Mahwah, NJ: Lawrence Erlbaum.

[B13] Boughton K. A. Yamamoto K. (2007). “A hybrid model for test speededness,” in *Multivariate and Mixture Distribution Rasch Models* eds von Davier M. Carstensen C. H. (New York: Springer) 147–156.

[B14] Bruno D. Rutherford A. (2010). How many response options? A study of remember-know testing procedures. *Acta Psychol.* 134 125–129. 10.1016/j.actpsy.2010.01.002 20137771

[B15] Burton R. (2001). Do item-discrimination indices really help us to improve our tests? *Assess. Eval. High. Educ.* 26 213–220. 10.1080/02602930120052378

[B16] Camilli G. (1993). “The case against item bias detection techniques based on internal criteria: Do test bias procedures obscure test fairness issues?,” in *Dierential Item Functioning* eds Holland P. W. Wainer H. (Hillsdale, NJ: Lawrence Earlbaum) 397–413.

[B17] Camilli G. Shepard L. A. (1994). *Methods for Identifying Biased Test Items.* Newbury Park, CA: Sage.

[B18] Chan D. Schmitt N. DeShon R. P. Clause C. S. Delbridge K. (1997). Reactions to cognitive ability tests: the relationships between race, test performance, face validity perceptions, and test-taking motivation. *J. Appl. Psychol.* 82 300–310. 10.1037/0021-9010.82.2.300 9109288

[B19] Cheung G. W. Rensvold R. B. (2000). Assessing extreme and acquiescence response sets in cross-cultural research using structural equations modeling. *J. Cross Cult. Psychol.* 31 187–212. 10.1177/0022022100031002003

[B20] Clemens N. H. Davis J. L. Simmons L. E. Oslund E. L. Simmons D. C. (2015). Interpreting secondary students’ performance on a timed, multiple-choice reading comprehension assessment: the prevalence and impact of non-attempted items. *J. Psychoeduc. Assess.* 33 154–165. 10.1177/0734282914547493

[B21] Crocker L. Algina J. (1986). *Introduction to Classical and Modern Test Theory.* New York, NY: Harcourt.

[B22] Culpepper S. A. (2015). Revisiting the 4-parameter item response model: bayesian estimation and application. *Psychometrika* 1–22. 10.1007/s11336-015-9477-6 26400070

[B23] DiBattista D. Kurzawa L. (2011). *Examination of the Quality of Multiple-Choice items on Classroom Tests.* Available at: http://ir.lib.uwo.ca/cgi/viewcontent.cgi?article=1061&context=cjsotl_rcacea

[B24] DiCerbo K. E. Oliver J. Albers C. Blanchard J. (2004). Effects of reducing attentional demands on performance of reading comprehension tests by third graders. *Percept. Motor Skills* 98 561–574. 10.2466/pms.98.2.561-574 15141921

[B25] Dorans N. J. Schmitt A. P. Bleistein C. A. (1992). The standardization approach to assessing comprehensive differential item functioning. *J. Educ. Meas.* 29 309–319. 10.1111/j.1745-3984.1992.tb00379.x

[B26] Eason S. H. Goldberg L. F. Young K. M. Geist M. C. Cutting L. E. (2012). Reader-text interactions: how differential text and question types influence cognitive skills needed for reading comprehension. *J. Educ. Psychol.* 104 515–528. 10.1037/a0027182 26566295 PMC4640191

[B27] Eid M. Rauber M. (2000). Detecting measurement invariance in organizational surveys. *Eur. J. Psychol. Assess.* 16 20–30. 10.1027//1015-5759.16.1.20

[B28] Embretson S. E. Reise S. P. (2000). *Item Response Theory for Psychologists.* Mahwah, NJ: Lawrence Erlbaum.

[B29] Emons W. H. M. (2009). Detection and diagnosis of person misfit from patterns of summed polytomous item scores. *Appl. Psychol. Meas.* 33 599–619. 10.1177/0146621609334378

[B30] Enders C. K. (2001). A primer on maximum likelihood algorithms available for use with missing data. *Struct. Equ. Modeling* 8 128–141. 10.1093/bioinformatics/btq651

[B31] Enders C. K. (2006). “Analyzing structural equation models with missing data,” in *Structural Equation Modeling: A Second Course* eds Hancock G. R. Mueller R. O. (Greenwich, CT: Information Age Publishing) 313–342.

[B32] Enders C. K. Peugh J. L. (2004). Using an EM covariance matrix to estimate structural equation models with missing data: choosing an adjusted sample size to improve the accuracy of inferences. *Struct. Equ. Modeling* 11 1–19. 10.1207/S15328007SEM1101_1

[B33] Furnham A. Hyde G. Trickey G. (2015). Personality and value correlates of careless and erratic questionnaire responses. *Pers. Individ. Differ.* 80 64–67. 10.1016/j.paid.2015.02.005

[B34] Gattamorta K. A. Penfield R. D. Myers N. D. (2012). Modeling item-level and step-level invariance effects in polytomous items using the partial credit model. *Int. J. Testing* 12 252–272. 10.1080/15305058.2011.630546

[B35] Geldhof G. J. Preacher K. J. Zyphur M. J. (2014). Reliability estimation in a multilevel confirmatory factor analysis framework. *Psychol. Methods* 19 72–91. 10.1037/a0032138 23646988

[B36] Glas C. A. W. (2009). What IRT can and cannot do. *Measurement* 7 91–93.

[B37] Gollwitzer M. Eid M. Jurgensen R. (2005). Response styles in the assessment of anger expression. *Psychol. Assess.* 17 56–69. 10.1037/1040-3590.17.1.56 15769228

[B38] Hambleton R. K. Swaminathan H. (1985). *Item Response Theory: Principles and Applications* Vol. 7 New York, NY: Springer

[B39] Hambleton R. K. Swaminathan H. Rogers H. J. (1991). *Fundamentals of Item Response Theory.* Newbury Park, CA: Sage Publications.

[B40] Han K. T. (2012). *Fixing the c Parameter in the Three-Parameter Logistic Model.* Available at: http://pareonline.net/getvn.asp?v=17&n=1

[B41] Harzing A. W. K. (2006). Response styles in cross-national survey research: a 26-country study. *Int. J. Cross Cult. Manag.* 6 243–266. 10.1177/1470595806066332

[B42] He J. van de Vijver F. (2012). Bias and equivalence in cross-cultural research. *Online Read. Psychol. Cult.* 2 1–19. 10.9707/2307-0919.1111

[B43] Holland P. W. Thayer D. T. (1988). “Differential item performance and the Mantel Haenszel procedure,” in *Test Validity* eds Wainer H. Brown H. I. (Hillsdale, NJ: Lawrence Erlbaum Associates) 129–145.

[B44] Hollman R. Glas C. A. W. de Haan R. J. (2003). Power analysis in randomized clinical trials based on item response theory. *Control Clin. Trials* 24 390–410. 10.1016/S0197-2456(03)00061-8 12865034

[B45] Huang J. L. Curran P. Keeney J. Poposki E. M. DeShon R. P. (2012). Detecting and deterring insufficient effort responding to surveys. *J. Bus. Psychol.* 27 99–114. 10.1007/s10869-011-9231-8

[B46] Johnson J. A. (2005). Ascertaining the validity of individual protocols from web based personality inventories. *J. Res. Pers.* 39 103–129. 10.1016/j.jrp.2004.09.009

[B47] Karabatsos G. (2003). Comparing the aberrant response detection performance of thirty-six person-fit statistics. *Appl. Meas. Educ.* 16 277–298. 10.1207/S15324818AME1604_2

[B48] Kurz T. B. (1999). A review of scoring algorithms for multiple-choice tests. *Paper presented at Annual Meeting of the Southwest Educational Research Association* San Antonio, TX 21–23.

[B49] Lau P. N. K. Lau S. H. Hong K. S. Usop H. (2011). Guessing, partial knowledge, and misconceptions in multiple-choice tests. *Educ. Technol. Soc.* 14 99–110.

[B50] Lepper M. R. Corpus J. H. Iyengar S. S. (2005). Intrinsic and extrinsic motivational orientations in the classroom: age differences and academic correlates. *J. Educ. Psychol.* 97 184–196. 10.1037/0022-0663.97.2.184

[B51] Lewandowski L. Cohen J. Lovett B. J. (2013). Effects of extended time allotments on reading comprehension performance of college students with and without learning disabilities. *J. Psychoeduc. Assess.* 31 326–336. 10.1177/0734282912462693

[B52] Liao W. W. Ho R. G. Yen Y. C. Cheng H. C. (2012). The four-parameter logistic item response theory model as a robust method of estimating ability despite aberrant responses. *Soc. Behav. Pers. Int. J.* 40 1679–1694. 10.2224/sbp.2012.40.10.1679

[B53] Linacre J. M. (1994). Sample size and item calibration stability. *Rasch Meas. Trans.* 7 328.

[B54] Linacre J. M. (2004). Discrimination, guessing and carelessness: estimating IRT parameters with rasch. *Rasch Meas. Trans.* 18 959–960.

[B55] Loken E. Rulison K. L. (2010). Estimation of a four–parameter item response theory model. *Br. J. Math. Stat. Psychol.* 63 509–525. 10.1348/000711009X474502 20030965

[B56] Lord F. M. (1974). Estimation of latent ability and item parameters when there are omitted responses. *Psychometrika* 39 247–264. 10.3389/fpsyg.2016.00255

[B57] Magis D. (2013). A note on the item information function of the four-parameter logistic model. *Appl. Psychol. Meas.* 37 304–315. 10.1177/0146621613475471

[B58] Maniaci M. R. Rogge R. D. (2014). Caring about carelessness: participant inattention and its effects on research. *J. Res. Pers.* 48 61–83. 10.1016/j.jrp.2013.09.008

[B59] Mantel N. Haenszel M. W. (1959). Statistical aspects of thee analysis of data from retrospective studies of disease. *J. Natl. Cancer Inst.* 22 719–748. 13655060

[B60] Meade A. W. Craig S. B. (2012). Identifying careless responses in survey data. *Psychol. Methods* 17 437–455. 10.1037/a0028085 22506584

[B61] Moors G. (2003). Diagnosing response style behavior by means of a latent-class factor approach: sociodemographic correlates of gender role attitudes and perceptions of ethnic discrimination reexamined. *Qual. Quant.* 37 277–302. 10.1023/A:1024472110002

[B62] Moors G. (2004). Facts and artifacts in the comparison of attitudes among ethnic minorities. A multigroup latent class structure model with adjustment for response style behavior. *Eur. Sociol. Rev.* 20 303–320. 10.1093/esr/jch026

[B63] Mroch A. A. Bolt D. M. Wollack J. A. (2005). A new multi-class mixture rasch model for test speededness. *Paper Presented at the Annual Meeting of the National Council on Measurement in Education* Montreal.

[B64] Oppenheimer D. M. Meyvis T. Davidenko N. (2009). Instructional manipulation checks: detecting satisficing to increase statistical power. *J. Exp. Soc. Psychol.* 45 867–872. 10.1016/j.jesp.2009.03.009

[B65] Pearson E. S. Hartley H. O. (1970). *Biometrika Tables for Statisticians* Vol. 1 3rd Edn. Cambridge: Cambridge University Press.

[B66] Penfield R. D. (2008). An odds ratio approach for assessing differential distractor functioning effects under the nominal response model. *J. Educ. Meas.* 45 247–269. 10.1111/j.1745-3984.2008.00063.x

[B67] Penfield R. D. (2011). How are the form and magnitude of DIF effects in multiple-choice items determined by distractor-level invariance effects? *Educ. Psychol. Meas.* 71 54–67. 10.1177/0013164410387340

[B68] Raiche G. Magis D. Blais J.-G. Brochu P. (2013). “Taking atypical response patterns into account: a multidimensional measurement model from item response theory,” in *Improving Large-Scale Assessment in Education* eds Simon M. Ercikan K. Rousseau M. (New York, NY: Routledge).

[B69] Raju N. S. van der Linden W. J. Fleer P. F. (1995). IRT-based internal measures of differential functioning of items and tests. *Appl. Psychol. Meas.* 19 353–368. 10.1177/014662169501900405

[B70] Rasch G. (1980). *Probabilistic Models for Some Intelligence and Attainment Tests.* Chicago: University of Chicago Press.

[B71] Raykov T. (2006). Interval estimation of optimal scores from multiple-component measuring instruments via SEM. *Struct. Equ. Modeling* 13 252–263. 10.1207/s15328007sem1302_5

[B72] Reckless G. E. Bolstad I. Nakstad P. H. Andreassen O. A. Jensen J. (2013). Motivation alters response bias and neural activation patterns in a perceptual decision-making task. *Neuroscience* 238 135–147. 10.1016/j.neuroscience.2013.02.015 23428623

[B73] Reise S. P. Waller N. G. (2003). How many IRT parameters does it take to model psychopathology items? *Psychol. Methods* 8 164–184. 10.1037/1082-989X.8.2.164 12924813

[B74] Reynolds N. Smith A. (2010). Assessing the impact of response styles on cross-cultural service quality evaluation: a simplified approach to eliminating the problem. *J. Serv. Res.* 13 230–243. 10.1177/1094670509360408

[B75] Robins J. Breslow N. Greenland S. (1986). Estimators of the mantel-haenszel variance consistent in both sparse data and large-strata limiting models. *Biometrics* 42 311–323. 10.2307/2531052 3741973

[B76] Rogers T. W. Bateson D. J. (1991). Verification of a model of test-taking behavior of high school seniors. *J. Exp. Educ.* 59 331–350. 10.1080/00220973.1991.10806571

[B77] Rulison K. Loken E. (2009). I’ve fallen and I can’t get up: can high-ability students recover from early mistakes in CAT? *Appl. Psychol. Meas.* 33 83–101. 10.1177/0146621608324023 20953275 PMC2954515

[B78] Rupp A. A. (2003). Item response modeling with BILOG-MG and MULTILOG for windows. *Int. J. Test.* 3 365–384. 10.1207/S15327574IJT0304_5

[B79] San Martin E. del Pino G. de Boeck P. (2006). IRT models for ability-based guessing. *Appl. Psychol. Meas.* 30 183–203.

[B80] San Martín E. González J. Tuerlinckx F. (2015). On the unidentifiability of the fixed-effects 3PL model. *Psychometrika* 80 450–467. 10.1007/s11336-014-9404-2 24482314

[B81] Sideridis G. D. Tsaousis I. Katsis A. (2014). An attempt to lower sources of systematic measurement error using hierarchical generalized linear modeling. *J. Appl. Meas.* 15 1–24. 25232668

[B82] Smith T. W. (2011). Refining the total survey error perspective. *Int. J. Public Opin. Res.* 23 464–484. 10.1093/ijpor/edq052

[B83] Swendsen J. D. (1998). The helplessness-hopelessness theory and daily mood experience: an idiographic and cross-situational perspective. *J. Pers. Soc. Psychol.* 74 1398–1408. 10.1037/0022-3514.74.5.1398

[B84] Swist K. (2015). Item analysis and evaluation using a four-parameter logistic model. *Edukacia* 3 77–97.

[B85] Thissen D. Steinberg L. (1986). A taxonomy of item response models. *Psychometrika* 51 567–577. 10.1007/BF02295596

[B86] Unlu A. (2006). Estimation of careless error and lucky guess probabilities for dichotomous test items: a psychometric application of a biometric latent class model with random effects. *J. Math. Psychol.* 50 309–328. 10.1016/j.jmp.2005.10.002

[B87] van der Linden W. J. (2007). A hierarchical framework for modeling speed and accuracy on test items. *Psychometrika* 72 287–308. 10.1007/s11336-006-1478-z

[B88] van Herk H. Poortinga Y. H. Verhallen T. M. M. (2004). Response styles in rating scales: evidence of method bias in data from six EU countries. *J. Cross Cult. Psychol.* 35 346–360. 10.1177/0022022104264126

[B89] Van Vaerenbergh Y. Thomas T. D. (2012). Response styles in survey research: a literature review of antecedents, consequences and remedies. *Int. J. Public Opin. Res.* 25 195–217. 10.1093/ijpor/eds021

[B90] Waller N. G. Reise S. P. (2009). “Measuring psychopathology with non-standard IRT models: fitting the four parameter model to the MMPI,” in *New Directions in Psychological Measurement with Model-Based Approaches* eds Embretson S. Roberts J. S. (Washington, DC: American Psychological Association) 147–173.

[B91] Ward M. K. Pond S. B. (2015). Using virtual presence and survey instructions to minimize careless responding on internet-based surveys. *Comput. Hum. Behav.* 48 554–568. 10.1016/j.chb.2015.01.070

[B92] Weijters B. (2006). *Response Styles in Consumer Research.* Doctoral dissertation, Ghent University Ghent.

[B93] Weijters B. Cabooter E. Schillewaert N. (2010a). The effect of rating scale format on response styles: the number of response categories and response category labels. *Int. J. Res. Mark.* 27 236–247. 10.1016/j.ijresmar.2010.02.004

[B94] Weijters B. Geuens M. Schillewaert N. (2010b). The individual consistency of acquiescence and extreme response style in self-report questionnaires. *Appl. Psychol. Meas.* 34 105–121. 10.1177/0146621609338593

[B95] Wen-Wei L. Rong-Guey H. Yung-Chin Y. (2012). The four-parameter logistic item response theory model as a robust method of estimating ability despite aberrant responses. *Soc. Behav. Pers.* 40 1679–1694. 10.2224/sbp.2012.40.10.1679

[B96] Woods C. M. (2006). Careless responding to reverse-worded items: implications for confirmatory factor analysis. *J. Psychopathol. Behav. Assess.* 28 189–194. 10.1007/s10862-005-9004-7

[B97] Wright B. D. Masters G. N. (1982). *Rating Scale Analysis.* Chicago, IL: MESA Press.

[B98] Wright B. D. Stone M. H. (1979). *Best Test Design.* Chicago, IL: MESA Press.

[B99] Yen Y. C. Ho R. G. Laio W. W. Chen L. J. Kuo C. C. (2012). An empirical evaluation of the slip correction in the four parameter logistic models with computerized adaptive testing. *Appl. Psychol. Meas.* 38 75–87. 10.1177/0146621611432862

