# Supplementary figures and images for: The Impact of Non-attempted and Dually-Attempted Items on Person Abilities Using Item Response Theory
Source: Front Psychol. 2016 Oct 14;7:1572. doi: 10.3389/fpsyg.2016.01572 (PMC5063855; doi:10.3389/fpsyg.2016.01572)

**Appendix A**

Two sample items (i1, i3) for Physics Subscale of the SAAT National Examination.


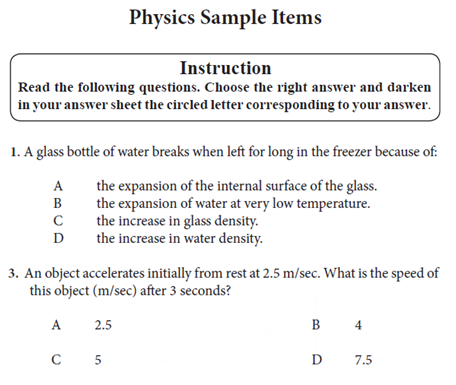

Supplement: Supplementary file 1 [file Data_Sheet_1.docx]
